# Broussochalcone A Induces Apoptosis in Human Renal Cancer Cells via ROS Level Elevation and Activation of FOXO3 Signaling Pathway

**DOI:** 10.1155/2021/2800706

**Published:** 2021-10-27

**Authors:** Han Ki Lee, Hyo Sun Cha, Myeong Jin Nam, Kyungmoon Park, Yung-Hun Yang, Jongsung Lee, See-Hyoung Park

**Affiliations:** ^1^Department of Biological Science, Gachon University, Seongnam 13120, Republic of Korea; ^2^Department of Bio and Chemical Engineering, Hongik University, Sejong 30016, Republic of Korea; ^3^Department of Biological Engineering, Konkuk University, Seoul 05029, Republic of Korea; ^4^Department of Integrative Biotechnology, Sungkyunkwan University, Suwon 16419, Republic of Korea

## Abstract

Broussochalcone A (BCA) is a chalcone compound extracted from the cortex of Broussonetiapapyrifera (L.) Ventenat that exerts various effects, such as potent antioxidant, antiplatelet, and anticancer effects. However, the effects of BCA against cancers have been seldom studied. This study is aimed at demonstrating the apoptotic mechanisms of BCA in A498 and ACHN cells, which are two types of human renal cancer cell lines. MTT, cell counting, and colony formation assays indicated that BCA treatment inhibited cell viability and cell growth. Further, cell cycle analysis revealed that BCA induced cell cycle arrest at the G2/M phase. Annexin V/PI staining and TUNEL assays were performed to determine the apoptotic effects and DNA fragmentation after treatment with BCA. Based on western blot analysis, BCA induced the upregulation of cleaved PARP, FOXO3, Bax, p21, p27, p53, phosphorylated p53 (ser15, ser20, and ser46), and active forms of caspase-3, caspase-7, and caspase-9 proteins, but downregulated the proforms of the proteins. The expression levels of pAkt, Bcl-2, and Bcl-xL were also found to be downregulated. Western blot analysis of nuclear fractionation results revealed that BCA induced the nuclear translocation of FOXO3, which might be induced by DNA damage owing to the accumulation of reactive oxygen species (ROS). Elevated intracellular ROS levels were also found following BCA treatment. Furthermore, DNA damage was detected after BCA treatment using a comet assay. The purpose of this study was to elucidate the apoptotic effects of BCA against renal cancer A498 and ACHN cells. Collectively, our study findings revealed that the apoptotic effects of BCA against human renal cancer cells occur via the elevation of ROS level and activation of the FOXO3 signaling pathway.

## 1. Introduction

Renal cell carcinoma (RCC) is a renal adenocarcinoma or a kidney cancer [1] and the most frequent type of kidney tumor found in mature individuals [2]. The kidney is a human organ that assists in waste removal while modulating fluid balance [3]. The kidneys contain small tubes, called tubules, that help to filter blood, assist with the excretion of feces, and promote the formation of feces [4]. RCC occurs when cancer cells grow uncontrollably in the inner wall of the renal tubules. Kidney cancer is a rapidly growing cancer that can easily metastasize to the lungs or surrounding organs. Currently, the incidence of RCC continues to increase at a rate of 2.5% every year [5]. Despite improvements in the effects of surgical methods and anticancer medicines in recent years, the prognosis of RCC remains poor [6]. To date, kidney transplantation has been an effective therapy for RCC patients. However, patients may need to take medicines for the remainder of their lives to avoid the refusal of the contributor's kidney by the body [7]. These antirejection agents thin the patient's bones and may cause adverse effects, such as diabetes and hypertension [8]. Therefore, it is necessary to develop alternative restorative strategies to treat renal cancer.

Traditional elements, such as flavonoids, have been identified as a crucial source of many drugs [9]. In particular, plant-derived compounds have been demonstrated as important materials for effective anticancer agents, such as vincristine, paclitaxel, and vinblastine [10]. Broussochalcone A (BCA) is a biologically active compound commonly found in Asian countries, such as Korea, China, and Japan. BCA is generally found in the cortex of Broussonetiapapyrifera (L.) Ventenat [11] and is known as a traditional medication for diuresis, hemostasis, and comfort against edema and cough. BCA is a dominant inhibitor of blood platelet cohesion [12] and an inhibitor of respiratory bursts in neutrophils [13]. Recent studies have demonstrated that BCA inhibits the orphan nuclear receptor, NR4A1, and induces apoptosis in pancreatic cancer MiaPaCa-2 and Panc-1 cells [14]. BCA has also been reported to have antitumor effects in human hepatocellular carcinoma HepG2 cells via the suppression of CYP2J2 [15]. However, the anticancer effects of BCA in human renal cancer cells have not been investigated.

Forkhead box O3 (FOXO3) belongs to the O subclass group of the forkhead family and serves as a transcription factor that modulates multiple physiological processes, such as cell cycle, endoplasmic reticulum (ER) stress response, metabolism, and cellular apoptosis [16]. FOXO3 is translocated from the nucleus to the cytoplasm following phosphorylation by the PI3K/Akt signaling pathway [17] and is related to many cancer cells as a modulator of apoptosis [18]. FOXO3 also plays a crucial role in cell cycle arrest [19]. Other studies revealed that FOXO3 regulated cell cycle arrest by enhancing the levels of p27 and reducing those of cyclin D [20]. Mitoxantrone treatment was found to significantly suppress phosphorylated Akt and cause the nuclear localization of FOXO3 in human osteosarcoma U2OS cells [21].

Gaining a better understanding of the molecular mechanisms underlying the process responsible for RCC development is of remarkable importance [22]. To identify a novel therapy against renal cancer, BCA was adopted as a FOXO3 activator in renal cancer cells based on the understanding of its apoptotic mechanisms. In our study, we aimed to determine the apoptotic effects of BCA in renal cancer A498 and ACHN cells. To demonstrate our therapeutic methods, we performed various apoptotic assays to detect the induction of apoptotic effects, including western blotting to identify variations in pro and antiapoptotic proteins.

## 2. Material and Methods

### 2.1. Chemical Reagents and Antibodies

BCA (purity, 95.8%) was purchased from the Institute for Korea Traditional Medical Industry (Daegu, Korea) and dissolved in DMSO (Sigma-Aldrich, St. Louis, MO, USA). A BCA stock solution of 40 mM was stored at -80°C. Mouse anti-*β*-actin (1 : 5000 dilution), rabbit anti-p-AKT (1 : 1000 dilution), rabbit anti-AKT (1 : 1000 dilution), rabbit anti-p-p53 (Ser15) (1 : 1000 dilution), rabbit anti-p-p53 (Ser20) (1 : 1000 dilution), rabbit anti-p-p53 (Ser46) (1 : 1000 dilution), and rabbit anti-MDM2 (1 : 1000 dilution) were purchased from Santa Cruz Biotechnology (Santa Cruz, CA, USA). Rabbit anti-p21 (1 : 1000 dilution), rabbit anti-p27 (1 : 1000 dilution), rabbit anti-p53 (1 : 1000 dilution), rabbit anti-FOXO3 (1 : 1000 dilution), rabbit anti-Bcl-2 (1 : 1000 dilution), rabbit anti-Bcl-xL (1 : 1000 dilution), rabbit anti-Bax (1 : 1000 dilution), rabbit anti-cleaved caspase-3 (1 : 1000 dilution), rabbit anti-caspase-3 (1 : 1000 dilution), rabbit anti-cleaved caspase-7 (1 : 1000 dilution), rabbit anti-caspase-7 (1 : 1000 dilution), rabbit anti-cleaved caspase-9 (1 : 1000 dilution), rabbit anti-caspase-9 (1 : 1000 dilution), rabbit anti-cleaved PARP (1 : 1000 dilution), and rabbit anti-PARP (1 : 1000 dilution) were purchased from Cell Signaling Technology (Danvers, MA, USA).

### 2.2. Cell Culture

Human renal cancer A498 and ACHN cells were purchased from the Korean Cell Line Bank (Seoul, Korea). The cells were maintained under standard conditions (5% CO2, 37°C, and 95% humidity). A498 cells were cultured in RPMI-1640 medium (GIBCO, Grand Island, New York, USA), and ACHN cells were cultured in DMEM (GIBCO) containing 10% heat-inactivated fetal bovine serum (Ab frontier, Korea) and 1% penicillin/streptomycin (GIBCO). Human embryonic kidney 293 T cells were purchased from American type culture collection (ATCC, Rockville, MD). The cells were maintained under standard conditions (5% CO2, 37°C, and 95% humidity). The cells were cultured in DMEM media (GIBCO) containing 10% heat-inactivated fetal bovine serum (Ab frontier, Korea) and 1% penicillin/streptomycin (GIBCO).

### 2.3. MTT Assay

A498, ACHN, and 293 T cells were seeded in 96-well plates at a density of 3 × 10^3^ cells per well and incubated overnight at 37°C in a humidified incubator containing 5% CO_2_. Thereafter, the cells were treated with different concentrations of BCA (0, 5, 10, 20, and 40 *μ*M). After 24 h, 48 h, and 72 h of treatment, 20 *μ*L of MTT dye (5 mg/mL) was added to each well, and the cells were incubated for 2 h at 37°C. The supernatants were subsequently removed, and the cells were treated with DMSO for formazan crystal formation. The plates were finally incubated on a shaker at room temperature (25°C) for 30 min, and the absorbance was measured at 570 nm using a spectrophotometer.

### 2.4. Cell Counting Assay

A498 and ACHN cells were seeded in 6-well plates at a density of 1 × 10^5^ cells per well and incubated overnight at 37°C in a humidified incubator containing 5% CO_2_. After incubation, the cells were treated with various concentrations of BCA (0, 10, and 20 *μ*M) for 24, 48, and 72 h and then stained with trypan blue solution. The cell numbers were counted using a hemocytometer.

### 2.5. Colony Formation Assay

A498 and ACHN cells (500 cells per well) were seeded in 6-well plates and incubated at 37°C in a humidified incubator containing 5% CO_2_ until stable. After the cells were treated with various concentrations of BCA (0, 5, 10, and 20 *μ*M) for 24 h, the medium was replaced with fresh media, and A498 and ACHN cells were incubated for 2 weeks under standard conditions. The cells were then rinsed twice with phosphate buffered saline (PBS) for 5 min each and fixed with 4% formaldehyde for 30 min at 4°C. Following fixation, the cells were washed twice with PBS for 3 min and stained with 1% crystal violet (Sigma-Aldrich) solution for 30 min. The number of colonies was then counted.

### 2.6. Cell Cycle Analysis

A498 and ACHN were seeded at a density of 2.5 × 10^5^ cells per well and incubated overnight at 37°C in a humidified incubator containing 5% CO_2_. After incubation, the cells were treated with various concentrations of BCA (0, 10, and 20 *μ*M) for 48 h. Following incubation, the cells were harvested by trypsinization and fixed in ice cold 70% ethanol overnight at 4°C. The cells were subsequently centrifuged at 1350 rpm for 5 min and incubated with PI working solution (Sigma-Aldrich; 50 *μ*g/mL PI and 200 *μ*g/mL RNase A) for 30 min at 37°C. Cell cycle distribution analysis was performed using flow cytometry (Beckman Coulter, Brea, CA, USA).

### 2.7. Annexin V/PI Staining Assay

A498 and ACHN cells were seeded in 6-well plates at a density of 2 × 10^5^ cells per well and incubated overnight at 37°C in a humidified incubator containing 5% CO_2_. After incubation, the cells were treated with various concentrations of BCA (0, 10, and 20 *μ*M) for 48 h, rinsed with PBS, and suspended in 1× binding buffer. The cells were stained with FITC-labeled annexin V and PI for 15 min at room temperature in the dark. After staining, the cells were analyzed by flow cytometry (Beckman Coulter, Brea, CA, USA).

### 2.8. TUNEL Assay

A498 and ACHN cells were seeded in 6-well plates at a density of 2 × 10^5^ cells per well and incubated overnight at 37°C in a humidified incubator containing 5% CO_2_. After incubation, the cells were treated with various concentrations of BCA (0, 10, and 20 *μ*M) for 48 h, rinsed twice with PBS for 5 min each, and fixed with 4% formaldehyde for 25 min at 4°C. Following fixation, the cells were rinsed twice with PBS for 5 min, permeabilized using Triton X-100 (0.2% PBS) for 10 min, and treated with 50 *μ*L of TdT enzyme buffer (equilibration buffer 45 *μ*L, nucleotide mix 5 *μ*L, TdT enzyme 1 *μ*L) for 1 h under standard conditions. Thereafter, 10 *μ*M Hoechst solution (in PBS) was added for 10 min, and DNA fragmentation was detected using a fluorescence microscope (Nikon Eclipse TE 2000-U, Tokyo, Japan).

### 2.9. Western Blot Analysis

A498 and ACHN were seeded in a 100 mm culture dish at a density of 1 × 10^6^ cells and incubated overnight at 37°C in a humidified incubator containing 5% CO_2_. After incubation, the cells were treated with various concentrations of BCA (0, 10, 20, and 40 *μ*M) for 24 h. All cells were collected in cold PBS, and lysates were harvested using radioimmunoprecipitation assay (RIPA) buffer (Cell Signaling Technology) containing protease inhibitor cocktail (Sigma Aldrich). After centrifugation, the supernatant was transferred to an EP tube. Protein concentrations were measured by the Bradford assay. The protein samples were separated via sodium dodecyl sulfate polyacrylamide gel electrophoresis at 120 V for 2 h. The proteins were transferred to a methanol-activated PVDF membrane at 25 V (4°C) overnight. The membranes were then blocked with 3% bovine serum albumin (BSA) for 30 min at room temperature and incubated overnight with the specific primary antibodies at 4°C. The membranes were washed three times with TBS-T every 10 min and incubated with a secondary antibody. Chemiluminescence was detected using a Chemi-doc detection system (Bio-Rad, Hercules, CA, USA).

### 2.10. Cytoplasmic and Nuclear Protein Fractionation

A498 and ACHN cells were grown in a 100 mm culture dish. At 70% confluency, the cells were treated with various concentrations of BCA (0, 10, 20, and 40 *μ*M) for 24 h and then fractionated using the Nuclear/Cytosol Fractionation kit (BioVision, Milpitas, CA, USA), according to the manufacturer's instructions with minor modifications. Cell pellets were obtained via centrifugation and washed with ice-cold PBS. The cells were then resuspended in 200 *μ*L of CEB-A (cytosolic extraction buffer A) and incubated on ice for 10 min. Subsequently, 11 *μ*L of CEB-B was added to the lysates, which were then vortexed and incubated on ice for 10 min. The cell lysates were centrifuged at 4°C for 5 min at 16,000 × g, and the supernatant was obtained as the cytoplasmic fraction. The cell pellets were washed with PBS, resuspended in NEB buffer, and incubated on ice for 10 min. Following incubation, sonication was performed to extract the nuclear protein. The lysates were centrifuged at 4°C for 10 min at 16,000 × g, and the supernatant was collected as the nuclear fraction. Both nuclear and cytoplasmic fractions were stored at -80°C.

### 2.11. DCF-DA Assay

A498 and ACHN cells were seeded in 60 mm cell culture dishes at a density of 2 × 10^5^ cells per well and incubated overnight at 37°C in a humidified incubator containing 5% CO_2_. After incubation, the cells were treated with BCA (0, 10, and 20 *μ*M) for 48 h. Additionally, cells were treated with or without 5 mM N-acetyl cysteine (NAC) as the control and 20 *μ*M of BCA. The cells were harvested by trypsinization, stained with 10 *μ*L of 20, 70-dichlorofluorescin diacetate (DCF-DA) solution in 4 mL of PBS for 30 min at room temperature (25°C), and analyzed by flow cytometry (Beckman Coulter Brea, CA, USA).

### 2.12. Comet Assay

A498 and ACHN cells were seeded in 60 mm cell culture dishes at a density of 2 × 10^5^ cells per well and incubated overnight at 37°C in a humidified incubator containing 5% CO_2_. After incubation, the cells were treated with BCA (0, 10, and 20 *μ*M) for 48 h, harvested by trypsinization, and resuspended in ice-cold PBS. The assay was performed using the Comet assay kit (Abcam ab238544), according to the manufacturer's instructions with minor modifications. The DNA-damaged cells were mixed with comet agarose in a 1/10 ratio (v/v). Thereafter, the slides were immediately blotted onto glass slides covered with a comet agarose base layer. After incubation with ice-cold lysis buffer, the slides were subjected to electrophoresis in a TAE electrophoresis solution. Subsequently, the slides were stained with Vista Green DNA dye. Damaged DNA was detected using a fluorescence microscope (Nikon Eclipse TE 2000-U, Tokyo, Japan).

### 2.13. siRNA Transfection

FOXO3-siRNA or control-siRNA was purchased from Santa Cruz Biotechnology (Santa Cruz, CA). The cells were transfected with FOXO3 or control siRNA using Lipofectamine 2000 reagent (Invitrogen) according to the manufacturer's protocol.

### 2.14. Statistical Analysis

The results are expressed as mean ± SEM (the standard error of the mean). To compare the statistical meaning between the groups, ANOVA followed by the Bonferroni posthoc test was used for statistical analysis, and a *p* value <0.05 was considered statistically significant. Experiments were repeated three times. And the representative data were shown.

## 3. Results

### 3.1. BCA Inhibits the Proliferation and Colony Forming Ability of A498 and ACHN Cells

**Figure 1:**
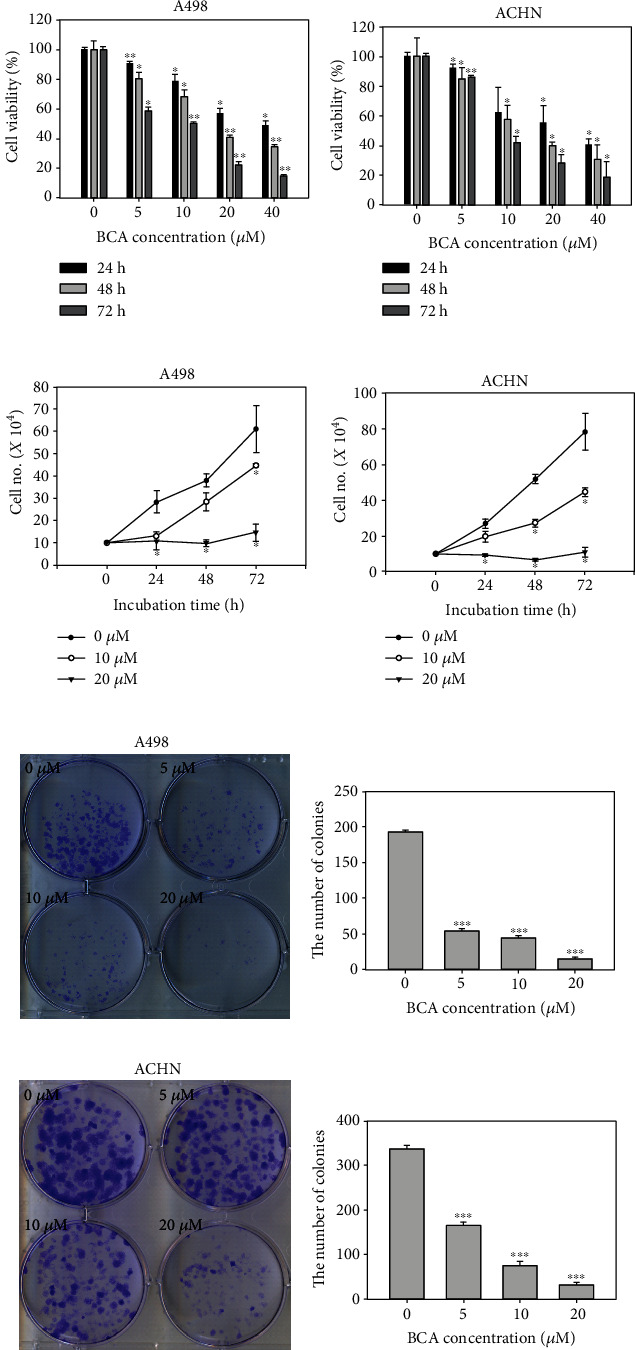
Antiproliferative effects of BCA against renal cancer cell lines. Dose- and time-dependent effects of BCA (0, 5, 10, 20, and 40 *μ*M) against (a) A498 and (b) ACHN cells after treatment of BCA for 24, 48, and 72 h. The cell viability was detected using the MTT assay. Cell counting assay of (c) A498 and (d) ACHN cells treated with BCA (0, 5, 10, and 20 *μ*M) for 24, 48, and 72 h. Colony formation assay of (e) A498 and (g) ACHN cells treated with BCA (0, 5, 10, and 20 *μ*M) following 2 weeks. (f, h) The error bar represented the standard error. Significant differences between BCA treat and DMSO control groups are indicated as ^∗^ (^∗^ means *p* < 0.05, ^∗∗^ means *p* < 0.01, ^∗∗∗^ means *p* < 0.005).

To determine the cytotoxic effects of BCA, we performed an MTT assay using the human renal cancer cell lines, A498 and ACHN. The MTT results revealed that the cytotoxic effect of BCA in A498 and ACHN cells occurred in dose- and time-dependent manners. A498 and ACHN cells were treated with various concentrations of BCA (0, 5, 10, 20, and 40 *μ*M) for 24, 48, and 72 h, respectively. After 48 h of treatment with 10 and 20 *μ*M BCA, the viability of A498 cells decreased to 68.3 and 40.96%, respectively. Under the same conditions, the viability of ACHN cells decreased to 57.86 and 40.12%, respectively (Figures 1(a) and 1(b)). 293 T cells were treated various concentrations of BCA (0, 5, 10, 20, and 40 *μ*M) for 72 h. 293 T cell viability decreased to 98.66, 93.29, 83.89, and 71.81% in a dose-dependent manner (Supplementary Figure 1). These results suggested that BCA has little effect on the viability of embryonic kidney 293 T cells compared to renal cancer cells. Based on the cell counting assay, BCA inhibited the growth of A498 and ACHN cells. After 48 and 72 h of incubation, the number of A498 and ACHN cells treated with BCA (20 *μ*M) was remarkably lower than that of cells treated with the control (DMSO) (Figures 1(c) and 1(d)). A colony formation assay was carried out to determine whether BCA suppressed the colony formation ability of A498 and ACHN cells. The results showed that BCA treatment markedly suppressed the colony formation ability of A498 (Figures 1(e) and 1(f)) and ACHN (Figures 1(g) and 1(h)) cells. Therefore, these results suggest that BCA displays a dominant inhibitory effect on the proliferation of renal cancer A498 and ACHN cells.

### 3.2. BCA Induces Cell Cycle Arrest in the G2/M Phase in A498 and ACHN Cells

**Figure 2:**
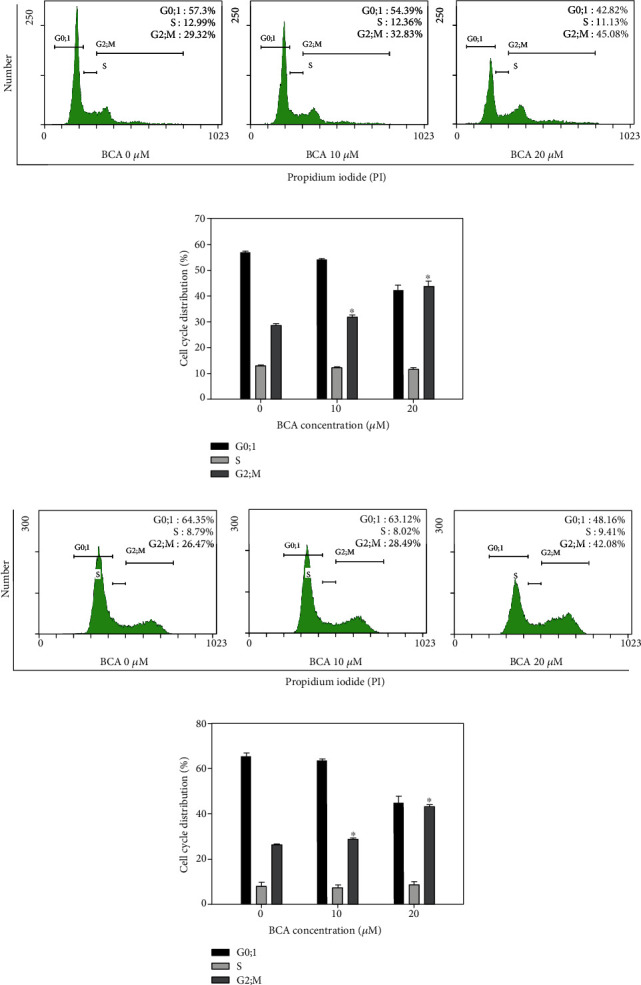
G2/M cell cycle arrest in renal cancer A498 and ACHN cells. A498 and ACHN cells were treated with 0, 10, and 20 *μ*M of BCA for 48 h and stained with propidium iodide. Following that, the cells were measured with FACS. The cytogram results showed that the cell distribution and percentage of cells in each proportion of the cell cycle were represented for (a) A498 and (c) ACHN cells. Distribution of (b) A498 and (d) ACHN cells of each proportion of the cell cycle was shown after BCA treatment. Significant differences between BCA treat and DMSO control groups are indicated as ^∗^ (^∗^ means *p* < 0.05).

To determine whether BCA induces cell cycle arrest in renal cancer cells, we performed cell cycle analysis using flow cytometry. An enhanced G2/M phase was found in both cells following BCA treatment in a dose-dependent manner. The G2/M population of A498 cells treated with 0, 10, and 20 *μ*M of BCA for 48 h was 29.32, 32.83, and 45.08%, respectively. The G2/M population of ACHN cells treated with 0, 10, and 20 *μ*M of BCA for 48 h was 26.47, 28.49, and 42.08%, respectively, as shown in Figure 2. Such findings indicate that BCA promoted G2/M cell cycle arrest in A498 and ACHN cells.

### 3.3. BCA Induces the Apoptosis of A498 and ACHN Cells

**Figure 3:**
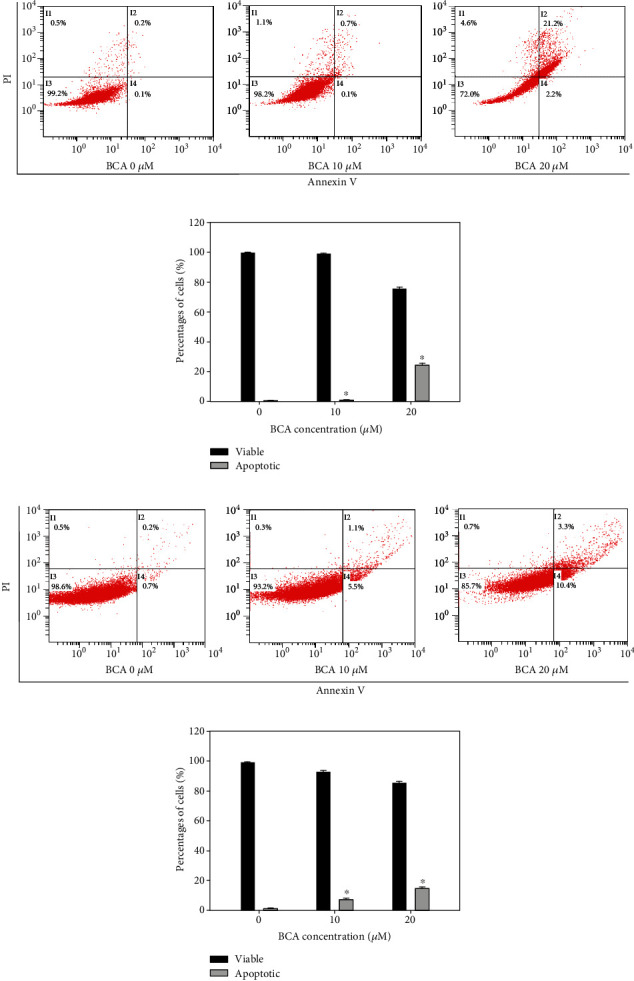
Induction of apoptotic cell death in A498 and ACHN cells by the annexin V and PI doubling staining assay following 48 h of treatment with BCA (0, 10, and 20 *μ*M). (a, c) The percentages of apoptotic cells against A498 and ACHN cells are represented in cytogram. (b, d) Percentages of viable and apoptotic cells were indicated in bar graphs. The error bar represented the standard error. Significant differences between BCA treat and DMSO control groups are indicated as ^∗^ (^∗^ means *p* < 0.05).

**Figure 4:**
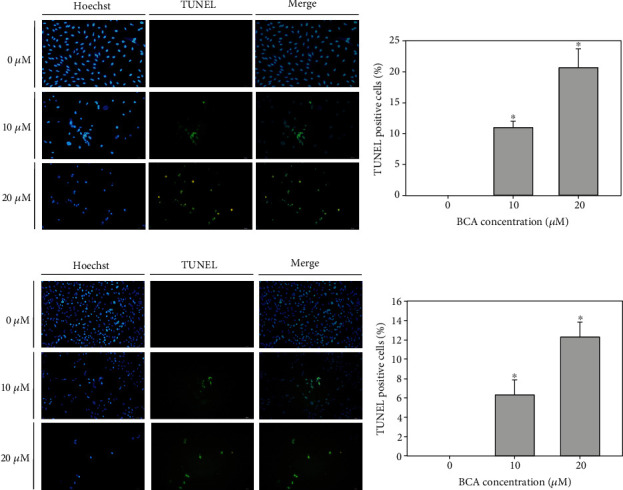
TUNEL assay results of A498 and ACHN cells treated with BCA (0, 10, and 20 *μ*M) for 48 h. DNA fragmentation was shown by fluorescence microscopy. (a, c) Blue fluorescence represents the nuclei stained using Hoechst 33342, and green fluorescence indicates the TUNEL-positive cells. (b, d) The bar graph showed that the percentages of TUNEL-positive cells after BCA treatment. The error bar represented the standard error. Significant differences between BCA treat and DMSO control groups are indicated as ^∗^ (^∗^ means *p* < 0.05).

We performed annexin V/PI double staining assays to investigate BCA-induced apoptotic cell death. The cytogram results revealed that the apoptotic cell death rate increased as the BCA concentration increased. The total apoptotic cell rates of A498 cells treated with 0, 10, and 20 *μ*M of BCA were 0.3%, 0.8%, and 23.4%, respectively (Figures 3(a) and 3(b)), while those of ACHN cells treated with the same concentrations of BCA were 0.9%, 6.6%, and 13.7%, respectively (Figures 3(c) and 3(d)). A TUNEL assay was performed to detect the fluorescence of apoptotic cells. The results showed that TUNEL-positive cells increased as the BCA concentration increased. Further, the percentages of TUNEL-positive cells in A498 cells treated with 0, 10, and 20 *μ*M of BCA were 0%, 11%, and 20.67% for 48 h, respectively (Figures 4(a) and 4(b)), while those of ACHN following treatment with BCA were 0%, 6.33%, and 12.33% for 48 h, respectively (Figures 4(c) and 4(d)). Overall, our results indicate that BCA treatment may induce cellular apoptosis and DNA fragmentation in renal cancer A498 and ACHN cells.

### 3.4. BCA Activates the Apoptotic Signaling Pathway in A498 and ACHN Cells

**Figure 5:**
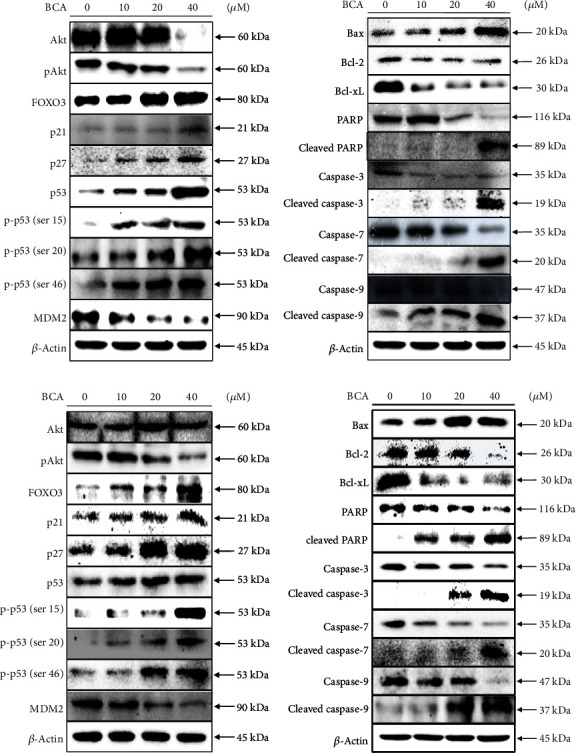
Western blot analysis of (a) A498 and (b) ACHN cells after treatment of BCA (0, 10, 20, and 40 *μ*M) for 24 h. The expression levels of specific proteins related to cell cycle arrest or apoptosis were detected by western blot analysis. *β*-actin was used as a loading control protein.

To demonstrate the apoptotic signaling pathways of BCA in A498 and ACHN cells, we conducted a western blot assay. We detected the expression levels of proteins related to cancer cell apoptosis and cell survival after BCA treatment. As shown in Figure 5(a) and 5(b), Akt protein levels were not affected by treatment with 20 *μ*M BCA; however, at 40 *μ*M BCA, its expression levels were decreased in A498 cells. In ACHN cells, Akt expression levels were not affected by BCA treatment. However, the level of phosphorylated Akt was decreased following BCA treatment in both cell lines. Treatment with BCA induced cleavage of poly (adenosine diphosphate-ribose) polymerase (PARP), caspase-3, caspase-7, and caspase-9, which are crucial regulators of apoptotic cell death in A498 and ACHN cells. In addition, the expression levels of proapoptotic proteins, such as Bax, were upregulated while those of antiapoptotic proteins, such as Bcl-2 and Bcl-xL, were downregulated. The expression levels of p21, p27, p53, and phosphorylated p53 (ser15, ser20, and ser46) associated with the cell cycle and tumor suppressor genes were found to increase with BCA treatment. However, the expression levels of MDM2 protein, also known as E3 ubiquitin-protein ligase, were decreased. BCA treatment induced the activation of FOXO3. In both cell lines, the expression levels of the FOXO3 protein significantly increased as the BCA concentration increased. Altogether, western blot analysis clarified the anticancer mechanisms of BCA. BCA treatment was found to activate the intrinsic apoptosis signaling pathways in A498 and ACHN cells.

### 3.5. BCA-Mediated Apoptosis Was Dependent on FOXO3 in A498 and ACHN Cells

**Figure 6:**
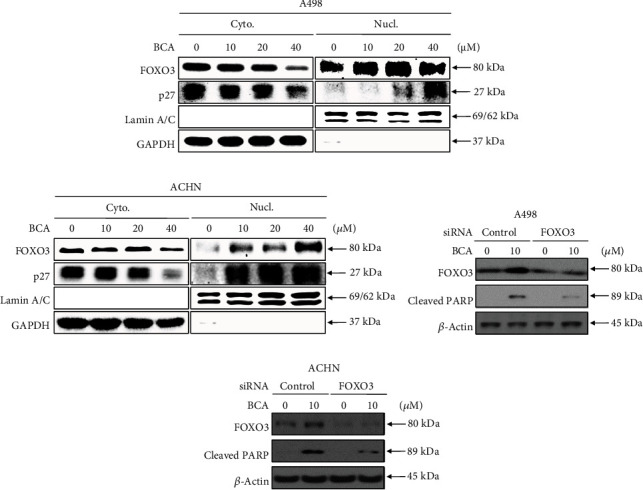
BCA induced the nuclear localization of FOXO3 protein and BCA-mediated apoptosis was dependent on FOXO3. Nuclear fraction western blot analysis results of (a) A498 and (b) ACHN cells treated with BCA (0, 10, 20, and 40 *μ*M) for 24 h. The expression levels of p27 and FOXO3 proteins in the cytoplasm and nucleus were investigated by performing western blot analysis. GAPDH, and Lamin A/C proteins were used for a gel-loading control for cytoplasmic and nuclear protein fractions, respectively. FOXO3 dependent apoptosis in renal cancer cells treated with BCA. Western blot results of (a) A498 and (b) ACHN cells transfected with control or FOXO3 siRNA and treated with BCA (0 and 10 *μ*M) for 24 h. *β*-actin was used as a loading control protein.

We performed nuclear fractional western blot analysis with lysates from A498 and ACHN cells to elucidate the anticancer mechanism of BCA. BCA treatment (0, 10, 20, and 40 *μ*M for 24 h) was found to decrease the expression levels of p27 and FOXO3 protein in the cytoplasm in a dose-dependent manner. However, the nuclear levels of FOXO3 and p27 proteins were significantly increased by BCA treatment in a dose-dependent manner (Figures 6(a) and 6(b)). These results suggested that BCA might induce the translocation of p27 and FOXO3 proteins from the cytoplasm to the nucleus in A498 and ACHN cells. To evaluate the role of FOXO3 in BCA-mediated apoptosis in renal cancer cells, we silenced the FOXO3 expression in A498 and ACHN cells and revealed the protein expression levels of PARP and FOXO3 using western blot assay. Interestingly, knockdown of the FOXO3 expression in A498 and ACHN cells attenuated the expression levels of the cleaved PARP and FOXO3 (Figures 6(c) and 6(d)). Taken together, these results suggested that FOXO3 might play a crucial role in accelerating the apoptosis after BCA treatment in A498 and ACHN cells.

### 3.6. BCA Increases Intracellular ROS Levels and Induces DNA Damage in A498 and ACHN Cells

**Figure 7:**
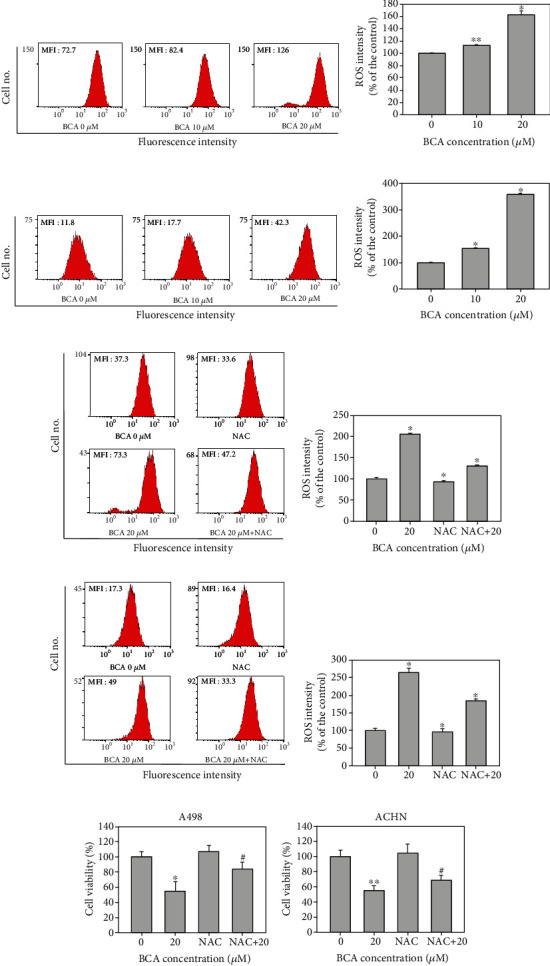
Measurement of ROS intensity in (a, e) A498 and (c, g) ACHN cells. Measurement of intracellular ROS intensity in BCA-treated A498 and ACHN cells cotreated with NAC also measured. (b, d, f, and h) The bar graph indicated that the percentage of control of MFI according to treatment of BCA and NAC. (i) The cell viability of A498 and ACHN cells in response to control, BCA, NAC, and cotreatment of BCA and NAC.^∗^indicated the *p* value between the control and BCA treatment group, and # indicated the *p* value between NAC and NAC/BCA cotreatment group. Significant differences between BCA treat and DMSO control groups are indicated as ^∗^ (^∗^ means *p* < 0.05, ^∗∗^ means *p* < 0.01).

**Figure 8:**
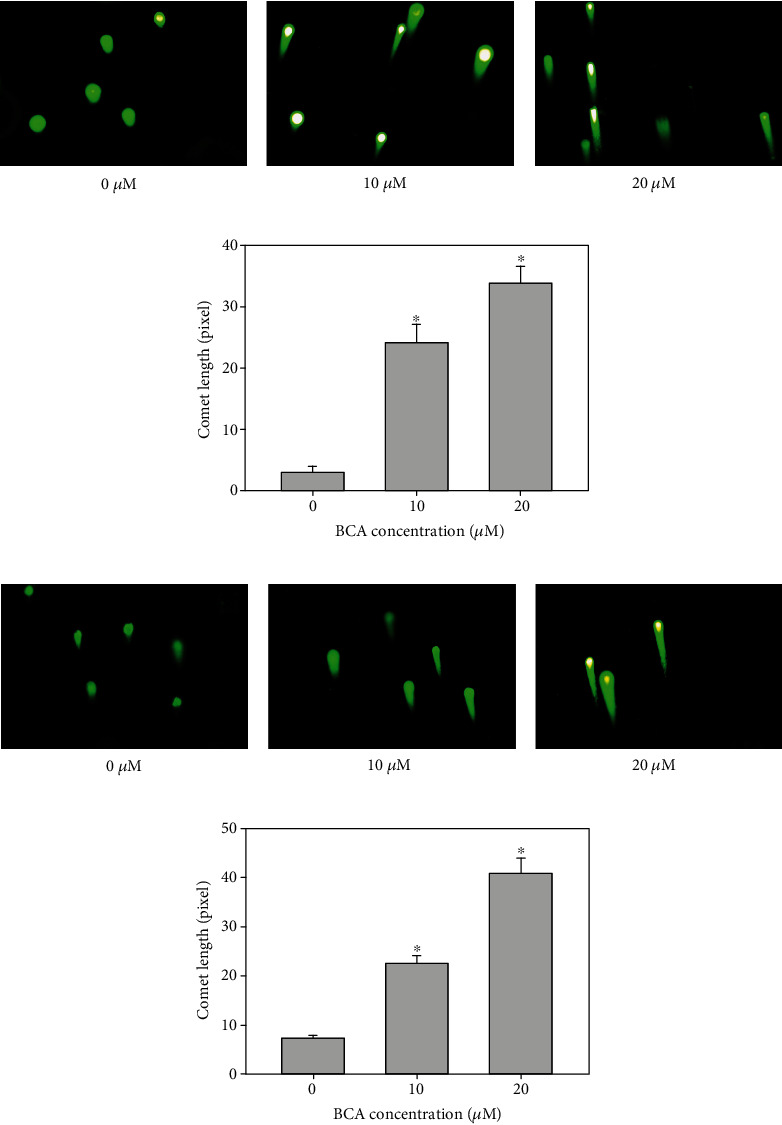
Comet assay was performed to identify BCA induces DNA damage in (a) A498 and (b) ACHN cells. The cells were treated with BCA (0, 10, and 20 *μ*M) of 48 h. BCA-induced DNA damage was investigated using a comet assay. As shown, the length of comet tail dose-dependently enhanced in response to (0, 10, and 20 *μ*M) BCA treatment. (c, d) The bar graph indicated the length of comet tail increased significantly. Significant differences between BCA treat and DMSO control groups are indicated as ^∗^ (^∗^ means *p* < 0.05).

We performed a DCF-DA assay to measure intracellular ROS levels in A498 and ACHN cells. DCF-DA analysis revealed that the mean fluorescence intensity (MFI) values of A498 cells treated with BCA (0, 10, and 20 *μ*M) were 72.7, 82.4, and 126, respectively (Figures 7(a) and 7(b)). Further, the MFI values of ACHN cells increased to 11.8, 17.7, and 42.3, respectively (Figures 7(c) and 7(d)). Treatment with BCA induced a significant increase of intracellular ROS generation in A498 and ACHN cells. ROS level was increased by 12.98% and 63.12% in A498 cells and 55.29% and 259.12% in ACHN cells, after 48 h of treatment with 10 and 20 *μ*M BCA, respectively. However, the MFI values were found to decrease when BCA-treated cells were cotreated with NAC. The cytogram results showed that the MFI values of the control, NAC, BCA, and NAC + BCA were 37.3, 33.6, 73.3, and 47.2 in A498 cells (Figures 7(e) and 7(f)) and 17.3, 16.4, 49, and 33.3, in ACHN cells (Figures 7(g) and 7(h)), respectively. The ROS generation in cells cotreated with NAC and BCA was decreased by 74.61% in A498 cells and 79.92% in ACHN cells compared to cells only treated with BCA for 48 h. Additionally, we measured the viability of cells cotreated with BCA and NAC using the MTT assay. Based on the results, the cell viabilities owing to the control, BCA, NAC, and NAC + BCA treatments were 100, 54.62, 107.23, and 83.96% for A498 cells and 100%, 55.32%, 104.26, and 68, 87% for ACHN cells, respectively (Figure 7(i)). These results suggest that BCA treatment induced the accumulation of ROS in human renal cancer cells, which might be associated with DNA damage in cells, thereby activating the apoptosis signaling pathways. Hence, we performed a comet assay to determine whether BCA induced DNA damage. As shown in Figure 8, the length of the comet tail of 0, 10, and 20 *μ*M of BCA was 3, 24.06, and 33.88 in A498 cells and 7.36, 22.64, and 40.79 in ACHN cells. Therefore, the length of the comet tail was found to increase as the BCA concentration increased. Taken together, these results suggest that BCA increased ROS and induced DNA damage in A498 and ACHN cells.

## 4. Discussion

Renal cell carcinoma (RCC) is a kidney cancer that develops within the inner barrier of the medial tubule, which is part of the highly thin tube of the kidney that carries primary urine [23]. RCC is the third most common type of cancer worldwide [24] and occurs more often in men than women (ratio of 1.5 : 1) [25]. RCC is also known to most often occur between the ages of 60 and 70 years [26]. To date, kidney transplantation and surgery are well-known treatments for RCC. However, these therapies can adversely affect the donor's body and lead to various side effects, such as the need to continue taking the medications to prevent rejection [7]. Recently, as drug-targeted therapeutics have been developed, cancer treatment methods have improved [27]. However, current treatment for advanced metastatic cancer is still accompanied by some adverse effects [28]. Therefore, to increase the effectiveness of cancer therapy and reduce the cost of cancer care, an appropriate treatment with less risk is required for more RCC patients [29]. Recently, chemical prophylaxis using natural flavonoids has emerged as a promising strategy for preventing renal cancer [30].

BCA is a natural flavonoid that is expected to play an important role in tumor therapy. In recent studies, BCA has been shown to exhibit therapeutic effects against various human diseases. BCA is a potent antioxidant inhibitor of detectable NO synthase against lipopolysaccharide-activated macrophages [31]. In addition, the inhibitory effects of BCA were investigated during the burst of respiration in neutrophils [13]. Recently, BCA was reported to be an inhibitor of the orphan nuclear receptor, NR4A1, and induces programmed cell death in pancreatic cancer cells [14]. Moreover, the cytotoxic effect of BCA against colorectal and liver cancer cells occurs via the accelerated destruction of complex-independent *β*-catenin degradation [32]. Furthermore, the inhibitory potential of BCA for the CYP2J2 isoform and its antitumor effects occur via FOXO3 activation [15]. However, the apoptotic effects of BCA against renal cancer cells have not been clearly revealed. In our study, we opted to demonstrate the apoptotic effects of BCA against human renal cancer A498 and ACHN cells.

Various apoptotic assays were performed to identify the apoptotic effects of BCA against several cancer cells. In a previous study, the inhibitory effects on cell proliferation, using WST-1, cell counting, and colony formation assays, revealed that BCA suppressed the survival of human hepatocellular carcinoma (HCC) HepG2 cells. In addition, sub-G1 cell cycle arrest was induced in HCC HepG2 cells by BCA treatment [15]. Our results, such as the MTT assay, cell counting, and colony formation assays (Figure 1), also showed that BCA inhibited the proliferation of renal cancer cells. Meanwhile, the results of cell cycle analysis revealed that G2/M cell cycle arrest was induced in renal cancer cells as BCA concentration increased (Figure 2). BCA has been reported to induce apoptosis in SW480, HCT116, and SNU475 cells [32]. Herein, apoptotic cell death was detected in both A498 and ACHN cells (Figure 3). Further, DNA fragmentation was detected in apoptotic cells following BCA treatment (Figure 4). Herein, we also demonstrated the anticancer mechanisms of BCA and detected variations in pro and antiapoptotic proteins through western blotting analysis.

Akt is a well-known oncogenic kinase that obstructs the tumor suppressive function of the FOXO3 transcriptional factor by promoting proteasome-mediated protein ubiquitination following phosphorylation [33]. Our western blot results showed that the Akt protein decreased when A498 cells were treated with 40 *μ*M of BCA; however, the levels of Akt did not appear to change significantly in ACHN cells after treatment with BCA (Figure 5). Nonetheless, phosphorylated Akt was found to decrease in both A498 and ACHN cells following BCA treatment. MDM2 protein, which promotes p53 protein deterioration and acts as an E3 ligase to p53, was significantly downregulated according to the progression of cancer cell apoptosis [34]. Our results revealed that the MDM2 protein expression was downregulated by BCA treatment in both cell lines. In addition, the expression levels of phosphorylated p53 (Ser15, Ser20, and Ser46) were markedly upregulated, which is thought to be a functionally active type of p53 that promotes apoptosis in cells [35]. We also measured the protein expression levels of p53 and found that the level of phosphorylated p53 (Ser15, Ser20, and Ser46) increased with BCA treatment.

Cancer cell apoptosis can be caused by intrinsic or extrinsic pathways [36]. Each pathway of cellular apoptosis is activated by diverse triggers, such as cell detachment, mitochondrial signals, and death ligands [37, 38]. Further, these pathways lead to caspase activation [39]. Our western blotting results showed that BCA stimulated caspase-9 in A498 and ACHN cells, which are the crucial initiators of caspase cascades in apoptotic cells [40]. Stimulation of caspase-9 caused the activation of caspase-3 and caspase-7 and led to cellular apoptosis in renal cancer cells.

The translocation of FOXO1 from the cytoplasm to the nucleus following inhibition of phosphorylated Akt occurs in U2OS cells stably expressing FOXO1 [41]. FOXO1 and FOXO3 are members of the FOX gene family, which involves a distinct forkhead DNA-binding domain [42]. Therefore, both of FOXO1 and FOXO3 proteins are well-known tumor suppressive transcriptional factors involved in many kinds of cellular functions, such as cell cycle arrest and apoptosis [43]. Thus, we performed nuclear fractional western blot analysis to confirm the translocation of FOXO3 into the nucleus, which causes an increase in the number of target genes and apoptosis [17]. Nuclear translocation of p27 was found to be induced by BCA treatment, which resulted in the inhibition of the cell cycle and induction of apoptotic cell death. The accumulation of cytoplasmic p27 might suppress apoptosis in cancer cells via its association with the activation of Akt, which is a typical suppressor protein of apoptotic cell death and inhibition of cytochrome release and caspase activation [44, 45]. Although these characteristics are present in the cytoplasm, p27 inhibits cyclin-dependent kinase 2 when localized in the nucleus [46]. Therefore, our results suggest that BCA can trigger the translocation of the FOXO3 protein from the cytoplasm to the nucleus in renal cancer cells (Figure 6).

FOXO3 activation is associated with ROS elevation and the induction of DNA damage [35, 47]. FOXO3 modulates the expression of proapoptotic proteins, such as Bax, and antiapoptotic proteins, such as Bcl-xL and Bcl-2. Based on our results, the expression levels of Bax protein increased and those of Bcl-xL and Bcl-2 decreased with BCA treatment (Figure 5). In a previous study, the induction of ROS-mediated ER stress was found to partially induce BCA-mediated apoptosis in pancreatic cancer cells. In addition, NAC was found to inhibit ROS elevation in pancreatic cancer cells [14]. Our results also demonstrated that BCA induced ROS accumulation in A498 and ACHN cells. However, NAC treatment inhibited ROS accumulation and promoted cell growth in A498 and ACHN cells (Figure 7). Herein, we evaluated DNA damage in A498 and ACHN cells using the comet assay. Accordingly, we found that the length of the comet tail increased with BCA treatment. Thus, these results suggest that BCA induces apoptosis via the activation of FOXO3 and causes ROS accumulation and DNA damage in A498 and ACHN cells.

In our study, we revealed the apoptotic effects of BCA in A498 and ACHN cells and its anticancer mechanisms. We elucidated the apoptotic activities of BCA, including the inhibition of cell proliferation and colony formation, G2/M cell cycle arrest, cellular apoptosis, ROS-mediated DNA damage, and translocation of FOXO3 protein into the nucleus in renal cancer cells. Therefore, we believe that the apoptotic effects of BCA could result from cell cycle arrest at the G2/M phase through the upregulation of FOXO3 and cell cycle alteration and proapoptotic proteins, such as p27 and Bax. Taken together, our results suggest that BCA treatment may induce FOXO3-mediated apoptosis in human renal cancer cells.

## Data Availability

The authors confirm that the data supporting the findings of this study are available within the article.

## Abbreviations

TUNEL: Terminal deoxynucleotidyl transferase dUTP nick end labeling
PARP: Poly (ADP-ribose) polymerase
Akt: Protein kinase B (PKB)
FOXO3: Forkhead box O-3
DMSO: Dimethyl sulfoxide
FBS: Fetal bovine serum
PBS: Phosphate-buffered saline
PI: Propidium iodide
DCF-DA: 2′,7′-Dichlorofluorescin diacetate
ROS: Reactive oxygen species
NAC: N-Acetyl cysteine.

## Supplementary Materials

Supplementary Materials Supplementary Figure 1: antiproliferative effects of BCA against 293 T cells. Dose-dependent effects of BCA (0, 5, 10, 20, and 40 *μ*M) against 293 T cells after treatment of BCA for 72 h. The cell viability was detected using the MTT assay. Significant differences between BCA treat and DMSO control groups are indicated as ^∗^ (^∗^ means *p* < 0.05, ^∗∗^ means *p* < 0.01). Supplementary Figure 2: graphical bar for western blot showed that the relative value of Akt, pAkt, FOXO3, p21, p27, p53, p-p53 (ser15, ser20, and ser46), MDM2, Bax, Bcl-2, Bcl-xL, PARP, cleaved PARP, caspase-3, cleaved caspase-3, caspase-7, cleaved caspase-7, caspase-9, and cleaved caspase-9 in (a) A498 cells and (b) ACHN cells. The relative expression levels were normalized to *β*-actin. Graphical bar for nuclear/cytosol fractionation western blot indicated that the relative value of FOXO3 and p27 in (c) A498 cells and (d) ACHN cells. The relative expression levels of cytosol were normalized to GAPDH, and the relative expression levels of nuclear were normalized to Lamin A/C. Graphical bar for FOXO3-siRNA transfection western blot showed that the relative value of FOXO3 and cleaved PARP in (e) A498 cells and (f) ACHN cells. The relative expression levels were normalized to *β*-actin.
