# Supplementary material for: Broussochalcone A Induces Apoptosis in Human Renal Cancer Cells via ROS Level Elevation and Activation of FOXO3 Signaling Pathway
Source: Oxid Med Cell Longev. 2021 Oct 27;2021:2800706. doi: 10.1155/2021/2800706 (PMC8566040; doi:10.1155/2021/2800706)
Supplement: Supplementary Materials — Supplementary Figure 1: antiproliferative effects of BCA against 293 T cells. Dose-dependent effects of BCA (0, 5, 10, 20, and 40 μM) against 293 T cells after treatment of BCA for 72 h. The cell viability was detected using the MTT assay. Significant differences between BCA treat and DMSO control groups are indicated as ∗ (∗ means p < 0.05, ∗∗ means p < 0.01). Supplementary Figure 2: graphical bar for western blot showed that the relative value of Akt, pAkt, FOXO3, p21, p27, p53, p-p53 (ser15, ser20, and ser46), MDM2, Bax, Bcl-2, Bcl-xL, PARP, cleaved PARP, caspase-3, cleaved caspase-3, caspase-7, cleaved caspase-7, caspase-9, and cleaved caspase-9 in (a) A498 cells and (b) ACHN cells. The relative expression levels were normalized to β-actin. Graphical bar for nuclear/cytosol fractionation western blot indicated that the relative value of FOXO3 and p27 in (c) A498 cells and (d) ACHN cells. The relative expression levels of cytosol were normalized to GAPDH, and the relative expression levels of nuclear were normalized to Lamin A/C. Graphical bar for FOXO3-siRNA transfection western blot showed that the relative value of FOXO3 and cleaved PARP in (e) A498 cells and (f) ACHN cells. The relative expression levels were normalized to β-actin. [file 2800706.f1.zip › 2800706_Supplemental Figures.docx]

**Supplementary Figures and Legends**


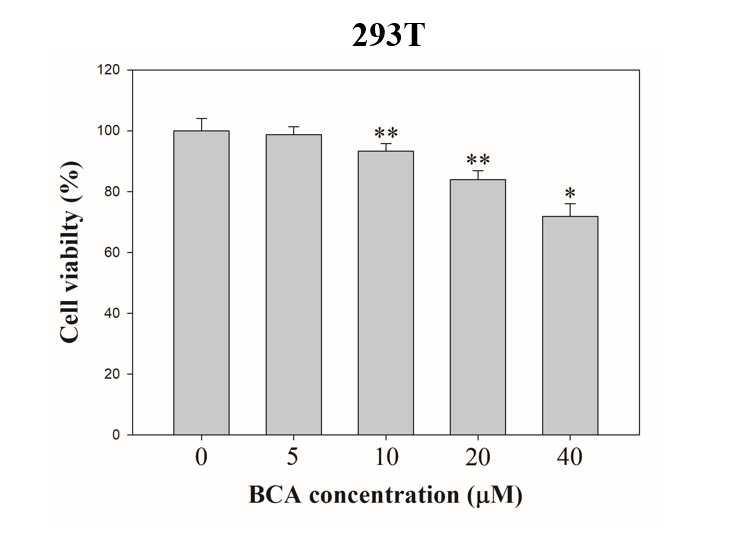


Supplementary Figure1. Antiproliferative effects of BCA against 293T cells. Dose-dependent effects of BCA (0, 5, 10, 20, and 40 μM) against 293T cells after treatment of BCA for 72 h. The cell viability was detected using MTT assay. Significant differences between BCA treat and DMSO control groups are indicated as * (* means p < 0.05, ** means p < 0.01).


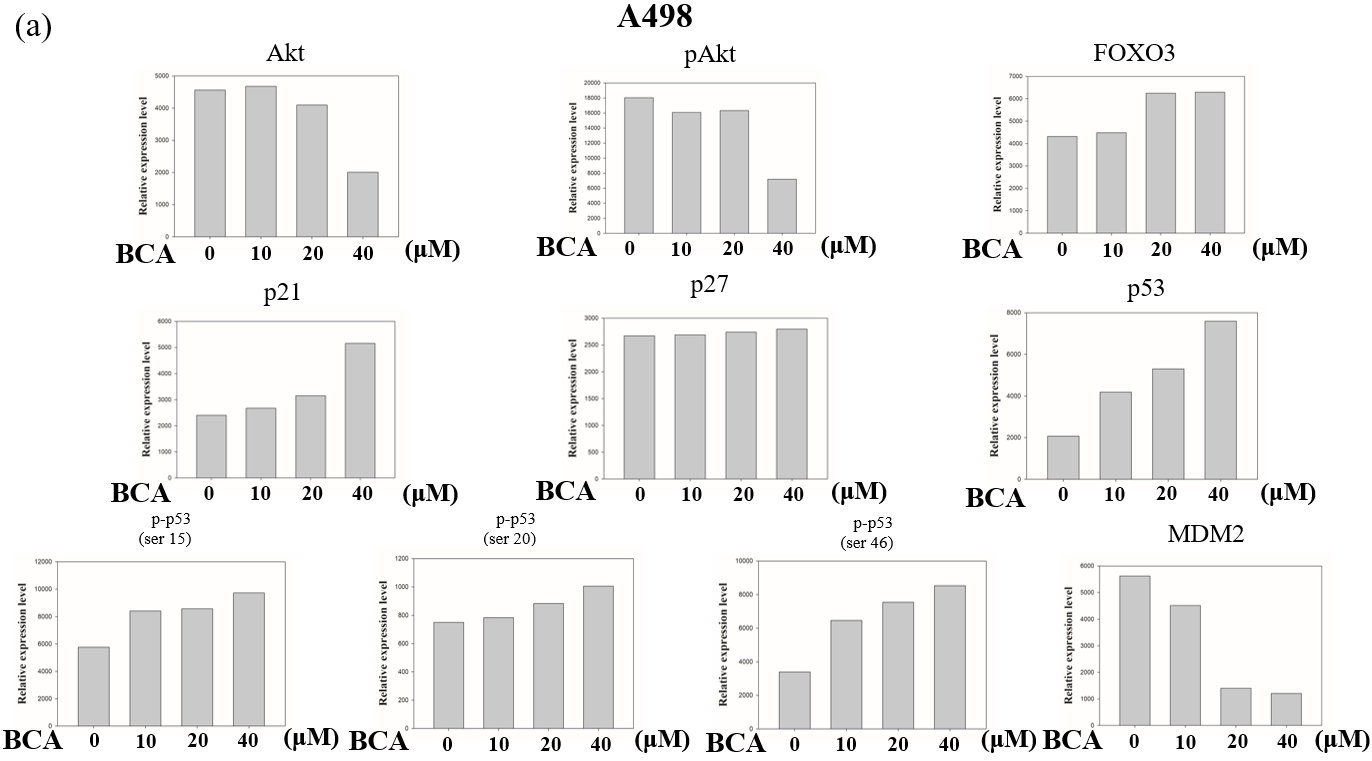

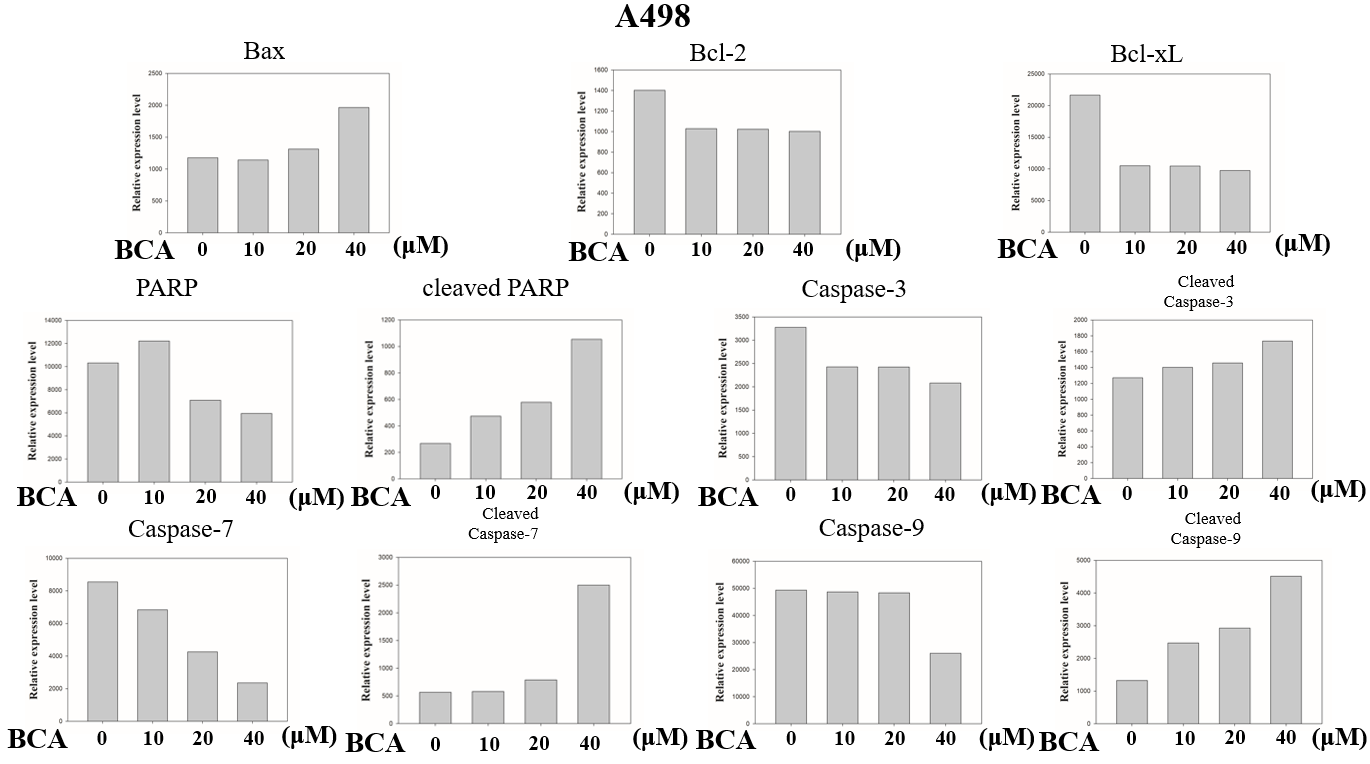

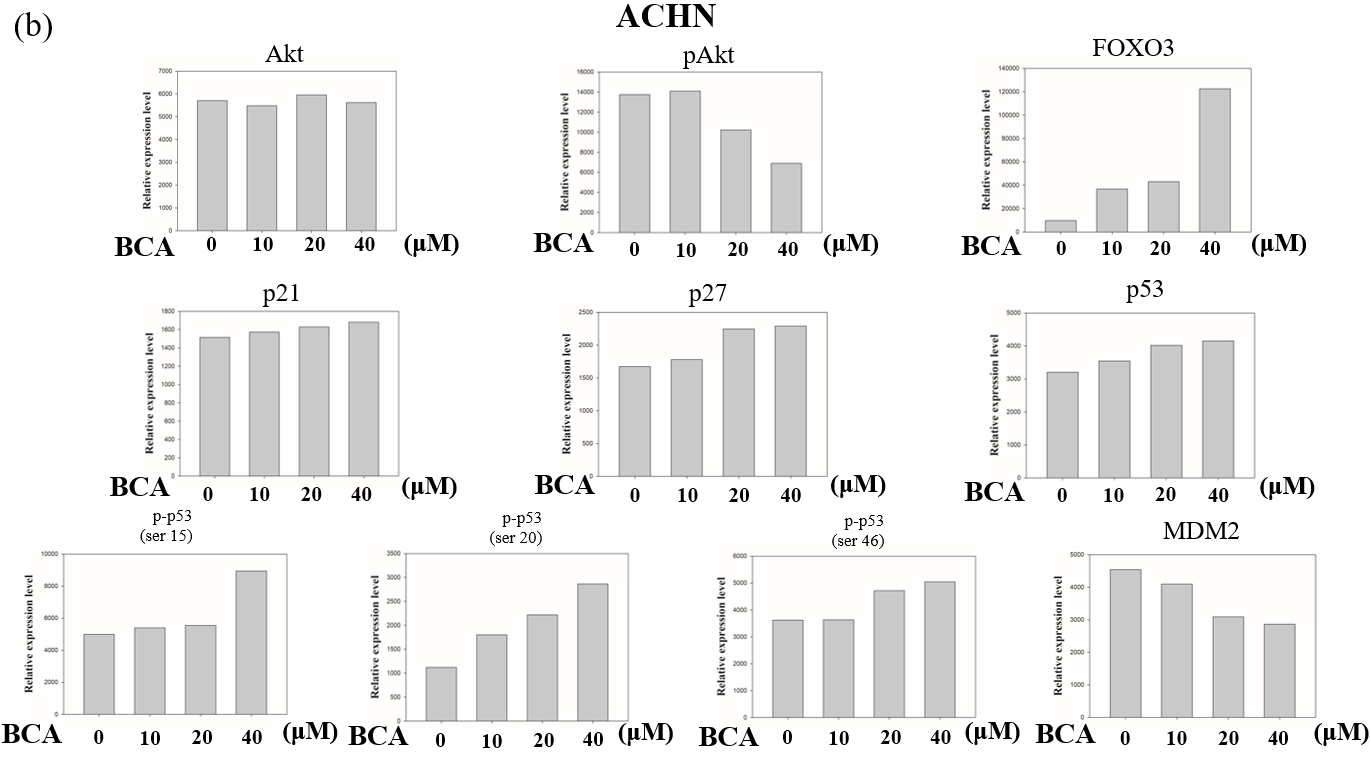

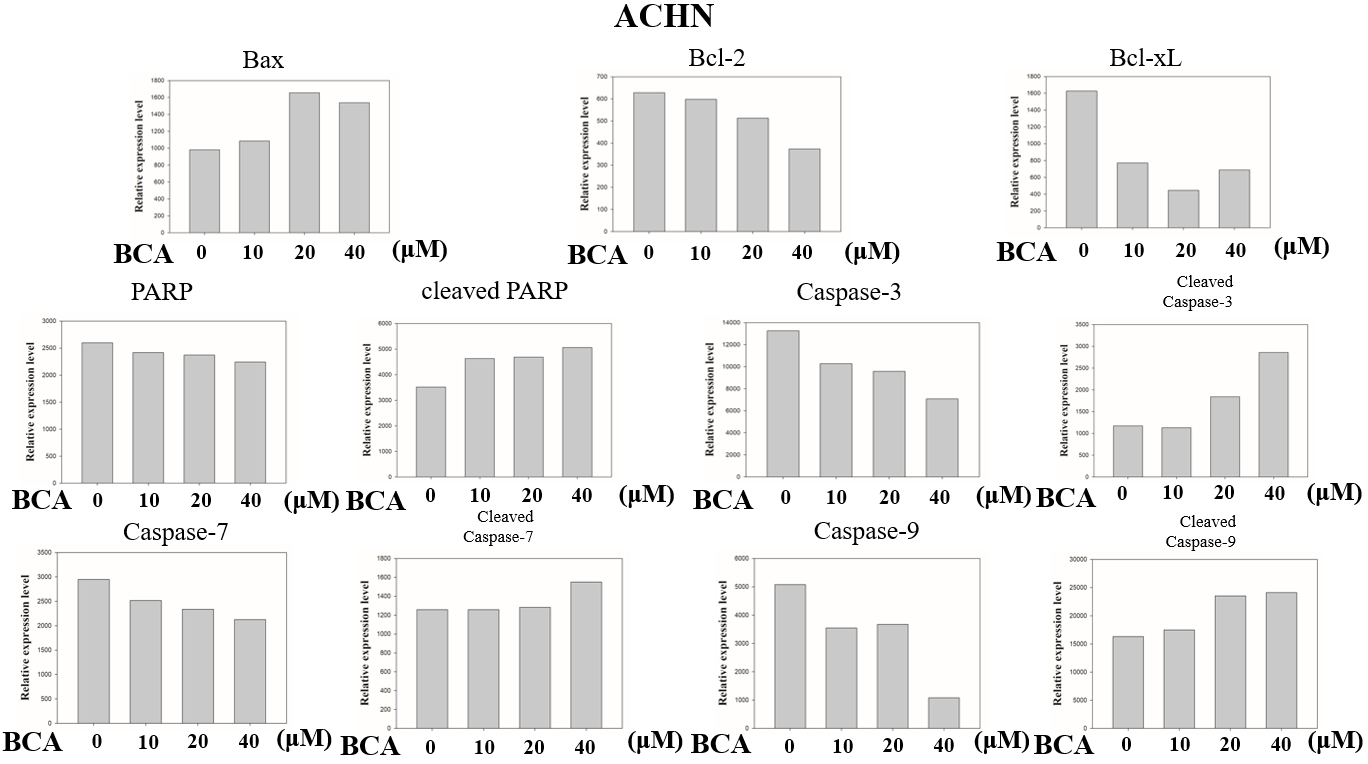

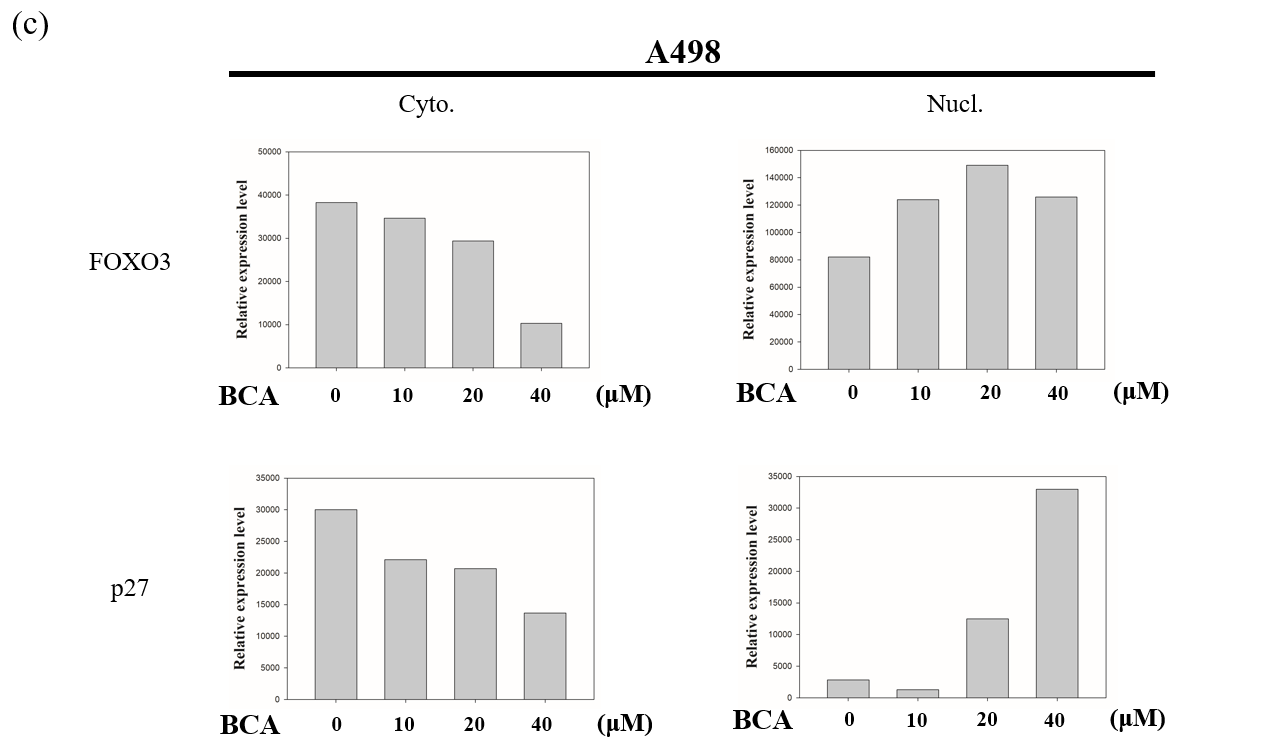

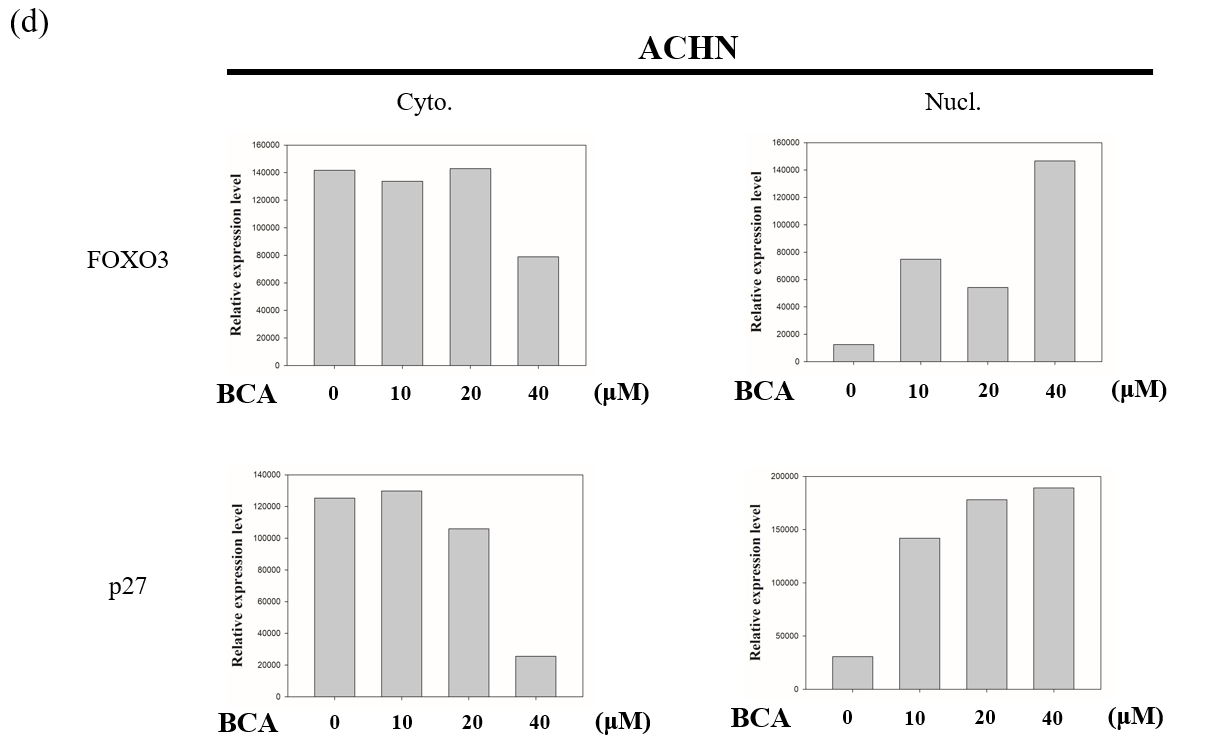

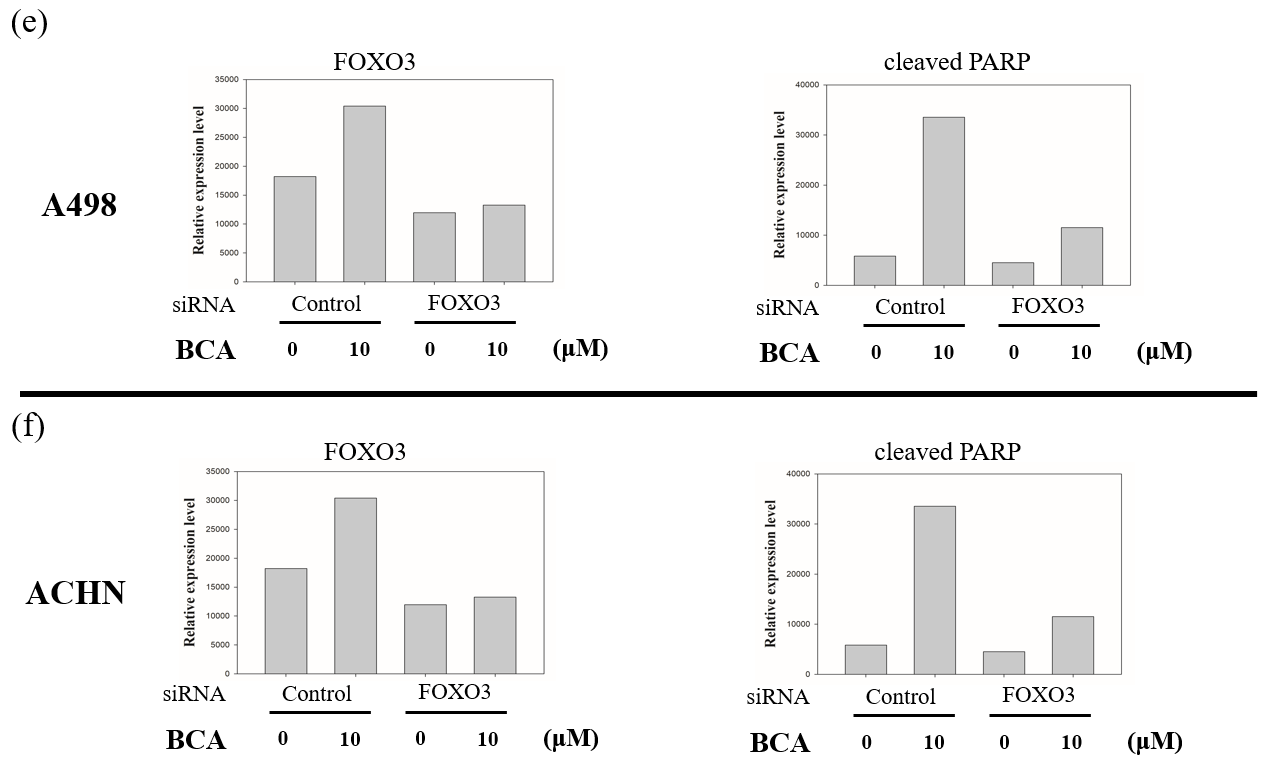


Supplementary Figure2. Graphical bar for western blot showed that the relative value of Akt, pAkt, FOXO3, p21, p27, p53, p-p53 (ser15, ser20, and ser46), MDM2, Bax, Bcl-2, Bcl-xL, PARP, cleaved PARP, caspase-3, cleaved caspase-3, caspase-7, cleaved caspase-7, caspase-9, and cleaved caspase-9 in (a) A498 cells and (b) ACHN cells. The relative expression levels were normalized to 𝛽-actin. Graphical bar for nuclear/cytosol fractionation western blot indicated that the relative value of FOXO3 and p27 in (c) A498 cells and (d) ACHN cells. The relative expression levels of cytosol were normalized to GAPDH and the relative expression levels of nuclear were normalized to Lamin A/C. Graphical bar for FOXO3-siRNA transfection western blot showed that the relative value of FOXO3 and cleaved PARP in (e) A498 cells and (f) ACHN cells. The relative expression levels were normalized to 𝛽-actin.
